# Editorial: Evolution and Functional Mechanisms of Plant Disease Resistance

**DOI:** 10.3389/fgene.2020.593240

**Published:** 2020-10-06

**Authors:** Jia-Yu Xue, Frank L.W. Takken, Madhav P. Nepal, Takaki Maekawa, Zhu-Qing Shao

**Affiliations:** ^1^Institute of Botany, Jiangsu Province and Chinese Academy of Sciences, Nanjing, China; ^2^College of Horticulture, Nanjing Agricultural University, Nanjing, China; ^3^Molecular Plant Pathology, Swammerdam Institute for Life Sciences, University of Amsterdam, Amsterdam, Netherlands; ^4^Department of Biology and Microbiology, South Dakota State University, Brookings, SD, United States; ^5^Department of Plant Microbe Interactions, Max Planck Institute for Plant Breeding Research, Cologne, Germany; ^6^School of Life Sciences, Nanjing University, Nanjing, China

**Keywords:** plant disease resistance, evolution, molecular mechanism, regulation mechanism, diversification

## Introduction

Plants in their natural environment are inevitably subject to diseases caused by pathogenic microbes and pests. To survive and combat these threats, plants have evolved a sophisticated immune system. The plant immune system comprises of two layers of defense (pathogen-associated molecular patterns -triggered immunity [PTI] and effector-triggered immunity [ETI]) that can be triggered by hundreds of immune receptors upon detection of pathogen-derived signatures. ETI is induced by resistance (R) proteins, whose activation typically results in death of infected cells (DeYoung and Innes, 2006). Many R proteins belong to the NBS-LRR/NLR (nucleotide-binding domain and leucine-rich repeats) class of intracellular receptors (Jacob et al., 2013). As *R* genes render great value to crop protection, exploration and identification of functional *R* genes has greatly expanded over the past 30 years, with >300 functional *R* genes isolated since 1992 (Kourelis and van der Hoorn, 2018). Besides significant progress in *R* gene cloning, functional studies revealed nine distinct modes of pathogen perception by *R* genes (Kourelis and van der Hoorn, 2018). *R* genes and pathogens are involved in a dynamic “arms race” (Yang et al., 2013), which can be costly for the plant (Tian et al., 2003). As R proteins can trigger cell death, their expression is tightly controlled at both the transcriptional and post-transcriptional level by e.g., methylation, miRNA, and phasiRNAs (Richard et al., 2018a). Despite the large progress made so far, there are many unanswered questions related to, for instance, evolution and regulation of *R* genes, their mode of action and how these proteins initiate downstream immune signaling.

This topic presents recent advances in plant disease resistance studies and the 14 publications include one review and 13 research articles, contributed by 122 authors. Based on a comprehensive review of the literature, Bayless and Nishimura summarized recent advances on TIR-domain containing R proteins, covering their evolution, structure, function, role in various cell death- and immune-pathways, and their downstream signaling partners. The 13 original articles can be broadly attributed to three topic areas.

## Resistance to Powdery Mildew in Wheat

Wheat is a globally important food crop. Three articles in this topic focus on resistance to powdery mildew in wheat. One study started with the confirmation of a single dominant gene (*PmJM23*) conferring mildew resistance. Subsequent bulked segregant RNA-Seq (BSR-Seq) analyses mapped the gene to the *Pm2* region on chromosome 5DS, where it could be narrowed down to six disease-related candidate genes (Zhu et al.) The other study revealed broad-spectrum resistance conferred by *PmJM23*, making it a useful source for resistance breeding. The linked markers were therefore used to screen breeding lines with high resistance to powdery mildew (Jia et al.) He et al. isolated 73 alleles of *Pm21*, another powdery mildew resistance gene from different wheat accessions, evaluated their genetic diversity, and discovered that the solvent-exposed LRR residues of proteins encoded by *Pm21* alleles had undergone diversifying selection. These studies will have implications in functional *R*-gene mining and utilization in wheat.

## Expression and Regulatory Mechanisms of Plant Resistance Signaling

When infected by pathogens, plant gene expression is extensively altered. Distinct transcriptome profiles emerge before- and after infection of resistant and susceptible plant lines. Li et al. compared the transcriptomes of resistant (*R*) and susceptible (*S*) rapeseed (*Brassica napus*) after inoculation with *Plasmodiophora brassicae* and observed differences between the *R*- and *S*-lines during early infection stages, revealing a quick plant response to the pathogen. A detailed transcriptome analysis on genes encoding a conserved protein family harboring the *D*omain of *U*nknown *F*unction 4228 (DUF4228) revealed that this gene family is highly responsive to infection by the fungal pathogen *Sclerotinia sclerotiorum* in *Arabidopsis thaliana*, castor bean and tomato. This finding suggests involvement of this uncharacterized protein family in disease resistance signaling to this devastating fungal pathogen (Didelon et al.) In wheat, small proteins were found to be specifically secreted into the apoplast upon infection with the fungal pathogen *Zymoseptoria tritici*. These proteins were experimentally verified to mediate recognition of pathogens and/or induce defense responses (Zhou B. et al.).

Expression of *R* genes is regulated by diverse factors (Richard et al., 2018a), such as small RNAs (Shivaprasad et al., 2012) and siRNAs affecting methylation of *NBS-LRR* genes (Richard et al., 2018b). In this topic, Zhang L.L. et al. discovered that a target mimic of miR156fhl-3p regulates miR156-5p and miR156-5p activity, which subsequently affect expression of *SPL14* and *WRKY45* enhancing rice blast disease resistance. Kong et al. showed that also DNA methylation is involved in regulating expression of *NBS-LRR* genes. Single, double and triple mutants of three *Arabidopsis* DNA demethylases (ROS1, DML2, and DML3) were found to affect CG methylation of specific *NBS-LRR* promoters and to alter their transcription, providing a new layer in the complex regulatory network controlling *NBS-LRR* activity (Kong et al.). Besides transcriptional control, the activity of *NBS-LRR* genes is regulated post-transcriptionally at the protein level. For a large extent the functionality of receptor NBS-LRR proteins is dictated by their interactions with other NBS-LRR (helper) proteins (Bonardi et al., 2011; Wu et al., 2018). In Solanaceae, a small family of the NLR Required for Cell Death (*NRC*) genes has been identified to be required for the function of NBS-LRR- and non-NBS-LRR immune sensors (Adachi et al., 2019). Notably, resistance conferred by many of these immune receptors is temperature sensitive and compromised above 28°C, implying that the helper NRCs could be the plant Achilles' heel at elevated temperatures. In this issue, it is shown that Rx1, an *NBS-LRR* gene from potato conferring resistance to Potato Virus X, which signals via NRC2, NRC3, or NRC4 (Wu et al., 2017), remains functional at high temperatures (Richard et al.). This finding implies that at least one helper NRC and its downstream signaling components are temperature tolerant, suggesting that thermosensitivity of the immune system is likely attributable to the receptors themselves.

## Evolution of *R* Genes

The past two decades witnessed a tremendous growth of studies focused on the abundance, origin, and evolution of the *NBS-LRR R* genes (Shao et al., 2019), facilitated by the advancement of information technology. Particularly, genome sequencing and bioinformatics tools integrated in molecular biology are increasingly providing novel insights into the evolution of the plant immune system. Four articles in the topic utilized rigorous bioinformatics and cutting-edge wet-lab experiments to identify- and characterize the *NBS-LRR* repertoire of 10 different plant species, including four species of orchids (Xue et al.), three species of soapberries (Zhou G.C. et al.), wheat (Andersen et al.) and African yam (Zhang Y.M. et al.). Among the ten species, hexapolyploid wheat has the highest number of *NBS-LRR* genes (>800) attributable to recent allopolyploidy events (Andersen et al.), while orchids have the lowest diversity attributable to gene-loss and less frequent gene duplication events (Xue et al.). In all species, most of the *NBS-LRR* genes were found to occur in gene clusters and a small portion of genes were singletons, whereas distinct expansion/contraction patterns were observed in the different species. Andersen et al. discovered that *R* protein gained integrated domains by alternative splicing, which allows creation of genetic diversity by *R* gene mRNAs that may encode for baits, decoys, and functional signaling components.

This Research Topic on Evolution and Functional Mechanisms of Plant Disease Resistance offers recent advances and insights in the field of plant disease resistance. We hope that with the rapid development of molecular technologies, analytical approaches, and methods, such as high-throughput sequencing, integrative multi-omics, and CRISPR gene editing, the reported advances help researchers to study and better understand Plant-Pathogen interactions.

## Author Contributions

J-YX compiled the contributions from all authors. All authors revised and approved the final version of the manuscript.

## Conflict of Interest

The authors declare that the research was conducted in the absence of any commercial or financial relationships that could be construed as a potential conflict of interest.
